# Chopstick technique versus cross technique in LESS hysterectomy (CCLEH study): a prospective randomized controlled trial

**DOI:** 10.1186/s13063-022-06650-w

**Published:** 2022-08-20

**Authors:** Yuya Dou, Li Deng, Shuai Tang, Yuanyang Yao, Xiaolong Liang, Qunying Hu, Yanzhou Wang

**Affiliations:** Department of Obstetrics and Gynecology, Southwest Hospital, Third Military Medical University, No. 30, Gaotanyan Street, Shapingba District, Chongqing, 400038 China

**Keywords:** Laparoendoscopic single-site surgery, Total hysterectomy, Cross technique, Chopstick technique

## Abstract

**Background:**

The traditional cross technique can be used to complete most laparoendoscopic single-site surgery (LESS) procedures, but some relatively precise operations, such as vaginal stump suturing, are challenging. In practice, we have introduced a novel technique named the chopstick technique and applied it to more complex operations, such as cervical cancer operations, and found that it contributes to performing delicate surgery. The efficacy and safety of two different surgical techniques in LESS hysterectomy remain to be validated.

**Methods:**

Patients who undergo total hysterectomy will be enrolled in this RCT. Stratified randomization will be performed according to uterine size (< 10 cm, 10–15cm, ≥ 15 cm). The participants will be divided into the chopstick technique group or cross technique group to undergo laparoendoscopic single-site total hysterectomy (LESS-TH), and then the perioperative and postoperative data, including the total operation time and other times, transfer rates, estimated blood loss, surgeon fatigue, intraoperative and postoperative complications (within 8 weeks after surgery), health-related quality of life (EQ-5D) scores, postoperative hospital stay, and hospitalization expenses, will be evaluated. The primary outcome is the operating time for total hysterectomy under LESS, and the other outcomes are secondary outcomes.

**Discussion:**

It is expected that the efficacy of the two techniques in LESS, the chopstick technique vs. the cross technique, will be compared and accumulate safety data on the new techniques will be accumulated.

**Trial registration:**

ChiCTR2000040843, registered on June 16,2020

Protocol version:

Version 2.0; Date: 2020.05.10

**Supplementary Information:**

The online version contains supplementary material available at 10.1186/s13063-022-06650-w.

## Background

Laparoendoscopic single-site surgery (LESS) is a favored alternative to traditional laparoscopy in minimally invasive surgery, with an esthetic advantage and can reduce postoperative pain from the incision as well as the use of analgesics [1, 2], which has been popular in cholecystectomy, nephrectomy, appendectomy, partial colectomy, and gynecological surgery [3–7].

All LESS operations are performed through a single puncture channel. Missing surgical triangles, instrument collisions, visual depth barriers, etc., are all problems that surgeons need to overcome. The “chopstick effect” is a dramatic description of the difficulties encountered in surgical operations with such close-range instrument layouts. To overcome the “chopstick effect,” surgeons have experimented with a variety of methods, of which front-bending instruments and the “cross technique” are widely used [8, 9]. Norihiko Ishikawa [10] specifically introduced the application of the “cross technique” for the first time in 2009; since then, the method has been mainstream. Boruta [11] used the “cross technique” to complete laparoendoscopic single-site radical hysterectomy in patients with cervical cancer. This surgical technique is shown in the LESS operation video, which is recommended by the American Association of Gynecologic Laparoscopists (AAGL): the surgeon is positioned on the left side of the patient, which is the same as in traditional laparoscopic surgery, the camera enters from the channel on the opposite side of the surgeon, and the surgical instruments enter from the channel on the same side as the surgeon. The left-hand instrument is mainly used for lifting and exposing, the right-hand instrument is mainly used for tissue separation and dissection, and the left-hand instrument makes a circular motion around the right-hand instrument. Because the core technique involves the crossing of two instruments around the umbilicus to complete the operation, this type of technique is called the “cross technique.” In clinical practice, we have explored another surgical technique that completely differs from the “cross technique” in terms of the position of the surgeon and the layout of the equipment. We named it the “chopstick technique” because it is similar to the manner in which Asians use chopsticks. We retrospectively analyzed 73 cervical cancer patients in which we applied the “chopstick technique” in LESS radical hysterectomy [12]. There were 72 successful cases that obtained the same clinical effectiveness as that of traditional multiple port laparoscopic surgery. The learning curve analysis among 44 cases of LESS cervical surgery showed that a significant reduction in operation time and complications could be achieved after performing 15 surgeries.

No comparative studies have been conducted to compare different surgical efficiencies between LESS techniques thus far. Therefore, this study intends to verify the value of the “chopstick technique” in LESS-TH by comparing surgical efficacy and surgeon fatigue.

## Methods

### Trial design

**Fig. 1:**
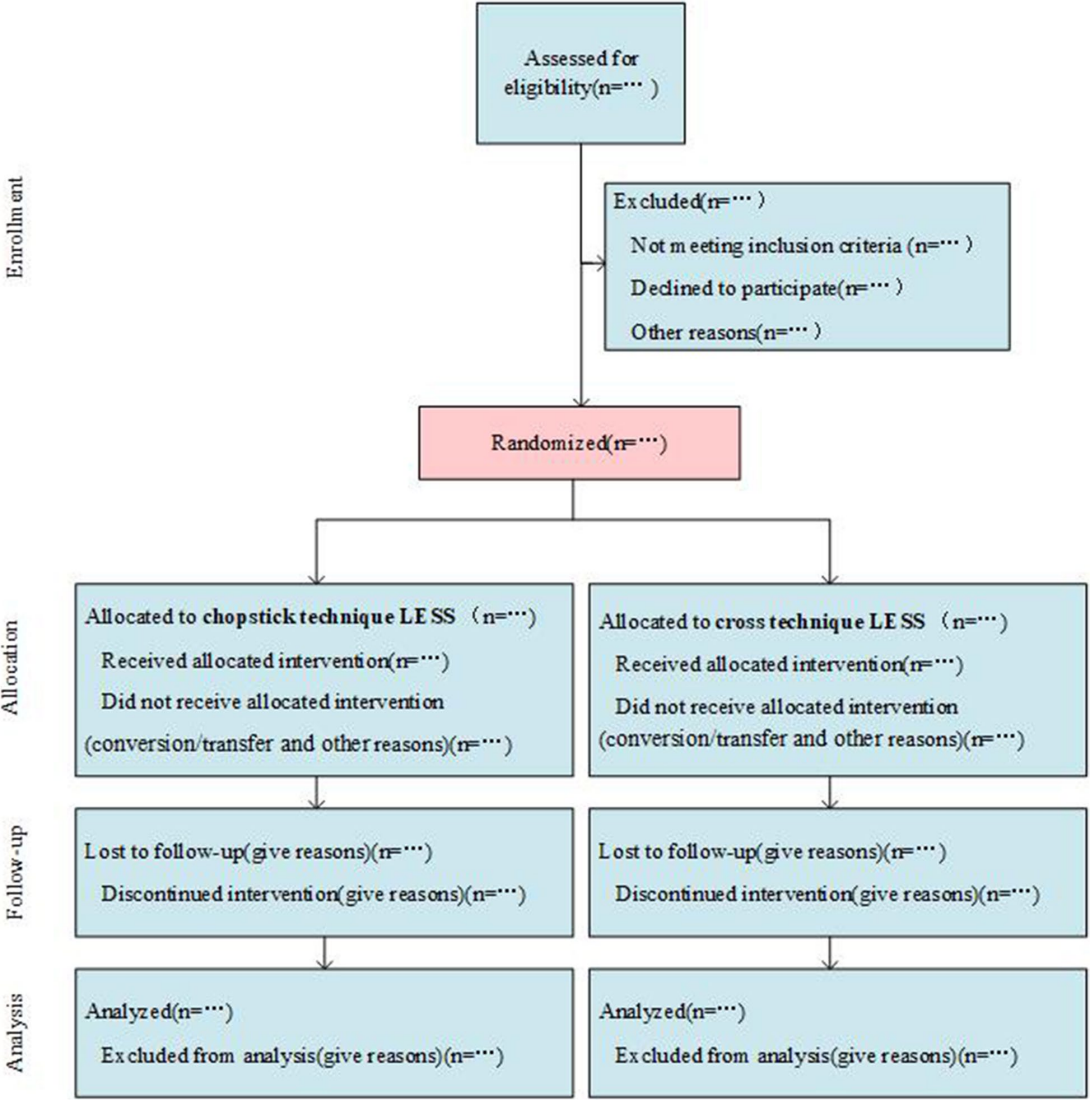
CONSORT flow diagram of the study. LESS, laparoendoscopic single-site injury

This single-center study will be a prospective, randomized controlled, double-blind, two-arm, parallel group, exploratory clinical trial carried out at the First Affiliated Hospital of the Third Military Medical University. The participant flow diagram is shown in Fig. 1.

The protocol complies with SPIRIT standards (http://www.spirit-statement.org/), which are recorded in detail in the SPIRIT checklist.

### Participants

The inclusion criteria are as follows:

1. Patients with indications for total hysterectomy, including uterine fibroids, adenomyosis, cervical precancerous lesions, endometrial atypical hyperplasia, IA1/LVSI-stage cervical cancer, early endometrial cancer (low-risk group), and Stage I endometrial stromal sarcoma
2. Patients with American Society of Anesthesiology (ASA) scores of 1–3
3. Patients aged 18–80 years

The exclusion criteria are as follows:

1. Other malignant tumors or the need to expand the scope of surgery
2. A plan for intraoperative freezing
3. Pelvic organ prolapse, vulvovaginal disease, and appendiceal disease
4. Suspected deep endometriosis
5. A history of pelvic or abdominal radiotherapy, peritoneal dialysis, pancreatitis, or pelvic tuberculosis
6. Surgical intolerance because of contraindications
7. Poor compliance
8. Refusal to provide informed consent

Patients will be stratified according to their uterine size without any exclusion regarding this aspect. The combination of salpingectomy or oophorectomy is also not an indication for exclusion since it makes no significant difference in the key operation steps and operation time.

### Interventions

1. Preoperative preparation

Patients will receive preoperative prophylactic antibiotics and will be given general anesthesia. A uterine manipulator will be placed.

2. Surgeon

All the surgeons participating in the study are proficient in the two LESS techniques (“chopstick technique” and “cross technique”) and have completed more than 40 operations using both surgical methods [13], which cumulatively exceeds the learning curve turning point.

3. Surgical treatment
  1. The position of the patient and the construction of a single-port channel

The patient is placed in the Trendelenburg position. A 2–2.5 cm longitudinal incision is made at the umbilicus layer-by-layer. The port incision protective sleeve is placed into the abdominal cavity and then tightened. The skin and the rectus abdominis are expanded and then connected with the port upper sealing cover, which forms a CO2 pneumoperitoneum.

2) The surgeon’s position and instrument layout

Experimental group: the “chopstick technique” group [12]

A. The position of the surgeons: The chief surgeon stands at the head of the patient, the camera assistant stands on the patient’s left, and the uterine-lift assistant stands between the patient’s legs.
B. The layout of the instruments: The camera and the operator’s two-handed instruments are arranged into a triangle, the camera is located at the top corner of the triangle, and the two operating instruments are located at the bottom corners (Table 1).
C. Operation technique: The surgeon’s hands are kept in a straight line with the instruments, and the operation skills are similar to those of traditional laparoscopy. The two instruments have independent fulcrums through the ports, which are not the fulcrums of each other.

**Table 1:**
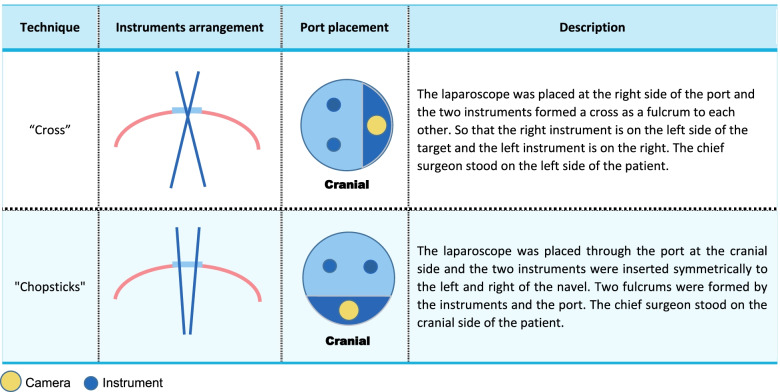
Characteristics and differences of the “cross” and “chopsticks” technique

Control group: the “cross technique” group

A. The positions of the surgeons: The chief surgeon stands on the patient’s left side, the camera assistant stands at the patient’s head, and the uterine-lift assistant stands between the patient’s legs.
B. The layout of the instruments: The camera enters from the channel on the opposite side of the surgeon, and the operation channels are all on the same side of the surgeon.
C. Operation technique: The left hand of the chief surgeon is mainly used for lifting and exposing, and the right hand is mainly used for tissue separation and cutting. The two instruments can cross each other through the ports to become each other's fulcrum, or the chief surgeon’s single-handed operation instrument and the assistant’s instrument form a cross fulcrum.
  3) Surgical steps

There are four steps to completed hysterectomy. Step 1: The periuterine peritoneum and superficial ligament are opened; the round ligament is cut off, the anterior broad ligament is opened, and the bladder and uterus reflexed peritoneum are separated. Step 2: The loose connective tissue around the uterus is separated, and the uterine arteries and veins are exposed and electrocoagulated. Step 3: A unipolar hook is used to remove the uterus along the fornix. Step 4: The vaginal stump is sutured with a barbed thread with a needle.

4) Conversion of the surgical method

Regardless of whether a patient is in the “chopstick technique” group or “cross technique” group, when the operation is difficult, whether it will be converted to the other LESS technique/multichannel laparoscopy/laparotomy is decided through evaluation by 2 gynecologists.

4. Postoperative care (under the enhanced recovery after surgery [ERAS] background)

Both groups will be nursed under the ERAS protocol [14]. Pain management will be standardized by the anesthesiologist. Patients will be encouraged to resume their diet and normal activities after surgery to facilitate functional recovery.

### Aims and objectives

Primary objective: the total operation time of the two LESS surgical techniques.

#### Secondary objectives

1. Single-port establishment time, pelvic-abdominal adhesion release time, total hysterectomy time (including salpingectomy or appendectomy time), specimen removal time, and vaginal stump suture time
2. Surgical conversion rate (switching to another LESS technique) and transfer rate (converting LESS surgery to traditional laparoscopy/laparotomy)
3. Estimated intraoperative blood loss
4. Intraoperative and postoperative complications (Clavien–Dindo grade≥ II级)
5. Score for health-related quality of life (EQ-5D)
6. Economic benefits with respect to hospitalization expenses
7. Postoperative hospital stay
8. Surgeon fatigue

### Sample size calculation

The sample size was calculated on the basis of the primary outcome, namely, the total operation time. Current reports on the “cross technique” for LESS-TH show that the average operation time is 170.1 ± 49.97 min [15]. According to our pilot retrospective study of 46 patients undergoing LESS-TH with the “chopstick technique,” the average operation time was 125.72 ± 51 min. The difference between the average values of the two operation time was determined as the minimum difference. A bilateral difference test was used. The first-order risk (*α*) is 0.05, and the power (1-β) is set at 0.9 for the study. Based on the evaluation of PASS 25.0 statistical software, each group requires 29 patients. The study consists of equivalent numbers for each treatment arm. In consideration of the loss to follow-up and multiple surgeons’ influences on the operation time, the sample size was expanded by 15% to 34 patients for each group. A total of 68 patients are required for the experiment.

### Randomization and blinding

In the study, stratified randomization will be adopted. Prior to randomization, all qualified participants will be classified according to uterine size by ultrasonic measurement (class A = uterine size smaller than 10 cm of the maximum diameter, class B = uterine size between 10 and 15 cm of the maximum diameter, and class C = uterine size larger than 15 cm of the maximum diameter), since uterine size is the primary determinant of the operation time for hysterectomy.

The research assistant will acquire a randomized timetable generated by a computer. Trial participants and research assessors will be blinded to the information of group allocation, as ensured by sequentially numbered, opaque and sealed envelopes. The research assistant will be excluded from the results evaluation and data collection. Patients will be blinded to distribution and intervention information. In case of serious adverse events, the outcome evaluator will inform the surgeon to evaluate whether it is related to the intervention and carry out further treatment without unblinding the patient.

### Recruitment and eligibility

Recruitment started on July 1, 2021. Patients who plan to undergo total hysterectomy will be enrolled.

Only qualified women who provide informed consent before randomization can be included in the trial. The recruitment of the required sample size (*N* = 68) can be completed within 1 year, followed by 6 months of follow-up and a 6-month analysis/reporting period, for a total of approximately 2 years for the whole process.

### Measurement and data collection

#### Outcome measures

The following baseline characteristics of patients will be documented: age, BMI, volume of the uterus, concomitant medications, EQ-5D scores, and anesthesiology scores (ASA).

B. The specific process for follow-up (Table 1):

During the screening period (before surgery), surgical indication, inclusion and exclusion indicators, gynecological check results will be investigated.

The collection of data on the day of surgery will include the successful removal of the uterus by the technique as randomized; conversion to laparoscopy or laparotomy; the total operation time (classified as the single-port establishment time, pelvic-abdominal adhesion release time, total hysterectomy time, specimen removal time, and stump suture time); estimated blood loss; intraoperative complications (including blood transfusion, vascular repair, bowel surgery, bladder surgery, ureter operation); and surgeon fatigue.

On the day of discharge, data on hospitalization expenses and the postoperative hospital stay will be collected by the research assessor.

During the 8 weeks after the intervention, postoperative complications (such as a second operation, infection, urogenital tract injury, intra-abdominal hemorrhage, incision hematoma, incision infection) will be recorded and rated by the Clavien–Dindo classification method. Health-related quality of life (EQ-5D) will be assessed again at 8 weeks (Table 2).

**Table 2:** Patient’s characteristics and data collection

Timepoint	Study period							
	Enrollment	Allocation	Post-allocation					Close-out
		0	Pre-operation	Operation	Post-operation	Discharge	After 8 weeks	
**Enrollment**								
Eligibility screen	X							
Informed consent	X							
Allocation		X						
**Interventions**								
Chopsticks technique				X				
Cross technique				X				
**Assessments**								
Baseline characteristics			X					
Total operation time^a^				X				
Single-port establishment time^b^				X				X
Pelvic-abdominal adhesion release time^c^				X				X
Total hysterectomy time^d^				X				X
Specimen removal time^e^				X				X
Stump suture time^f^				X				X
Conversion to another LESS technique				X				X
Conversion to traditional laparoscopy /laparotomy				X				
Estimated blood loss^f^				X	X			X
Intraoperative complications				X	X			X
Surgeon fatigue (FSS)^h^					X			X
Post-operative hospital stay						X		X
Hospitalization expenses						X		X
Post-operative hospital stay						X		
Postoperative complications^i^							X	X
Health-related quality of life (EQ-5D)			X				X	X

^a^ Total operation time: the time from the beginning of the skin incision to the end of the operation

^b^ Single-port establishment time: the time from the beginning of the skin incision to the establishment of pneumoperitoneum

^c^ Pelvic-abdominal adhesion release time: the time taken to separate adhesions

^d^ Total hysterectomy time: the time from accessory/fallopian tube resection to uterine disconnection

^e^ Specimen removal time: the time taken to remove the uterus through vaginal or umbilical fragmentation

^f^ Stump suture time: the time taken for vaginal stump suturing with a single needle barb

^g^ Estimated blood loss: collected and evaluated by the intraoperative suction device

^h^ Fatigue Severity Scale (FSS): score on a 7-point scale (1 = strongly disagree, 7 = strongly agree), where a higher mean value manifests a higher degree of severity of fatigue symptoms [16]

^i^ Postoperative infection: lower abdominal pain with fever>38°C

We will not involve participant biological samples.

### Data collection and management

The research assessor will record participant data in a case report form (CRF), which will be identified by the number and initial letters of the participants’ names without disclosing their information. Two data managers will independently input and proofread two copies, and establish a limited-access database that conducts computerized editing checks and manual checks.

### Statistical analysis

The research assistants will send nonblinded data to a statistician for analysis, who will remain blinded to the other investigators until all data analysis is complete. The data will be analyzed with SPSS version 25.0 software. Continuous variables that are normally distributed will be analyzed according to the means and SDs, and discrete variables that are nonnormally distributed will be summarized as medians, ranges, and IQRs. The *T* test and nonparametric analysis will be applied to analyze continuous variables with normal and nonnormal distributions, respectively, and categorical variables will be analyzed by the *χ* ^2^ test or Fisher’s exact test. *P* < 0.05 will indicate a statistically significant difference.

If it is limited to a single missing item, the missing data will be estimated from the given mean values. Imputation will not be attempted if the missing data include the whole table or more than one item. A sensitivity analysis will be performed to determine whether the method used to deal with missing data is appropriate.

According to the analysis of 1534 laparoscopic total hysterectomies by the Netherlands prospective cohort study [17], the surgical time is influenced by the uterine weight and the methods of hysterectomy, and the independent risk factors for surgical conversion are body mass index (BMI), uterine weight, laparoscopic hysterectomy method, and age. The stratified randomization of uterine weight will be conducted. Moreover, the BMI and age subgroups will be evaluated.

### Populations for the analyses

The analysis will first be conducted on the basis of the “intention to treat” principle. When patients who seriously violate the protocol (e.g., no objective postinclusion data) are observed, such data will be excluded.

### Monitoring

In the course of the investigation, a surgical video of each operation will be retained for use by the clinical trial independent data monitoring committee (DMC) for quality control review of the operation technique and the conversion of the surgical method. Given the limited resources and the single center design, there will be no auditing and interim analyses of the conduct of the trial.

### Harm

It is considered that this study does not increase any specific risk for the participants beyond those of laparoscopic surgery in general, especially when the surgeon does not hesitate to convert to traditional laparoscopy or laparotomy surgery. All adverse events will be recorded, including those spontaneously reported by participants and observed by investigators, and the relevance to the study will be identified. The investigator will provide corresponding compensation for research-related damage in accordance with the provisions of relevant laws. Serious adverse events must be reported to the ethics committee within 24 h.

### Legal aspect

The sponsor for this project is represented by the First Affiliated Hospital of Army Medical University and will provide insurance for the duration of the study.

### Patient and public involvement statement

Women who plan to undergo total hysterectomy will be randomly divided into an experimental group (chopstick technique group) and a control group (cross technique group) after providing informed consent. Both surgical techniques are common routine LESS techniques. We will observe the following indicators during hospitalization and follow-up: operation time, blood loss, intraoperative and postoperative complications, hospitalization expenses, hospitalization days, etc. Participants will be required to visit the hospital at the follow-up time, which is very important. There will be a call to remind participants of their follow-up. Their participation in this research will help to carry out the clinical application research and establish the technical specification for treatment. The “chopstick technique” is a local technique, and its superiority proves that it will help us to improve our international academic influence.

## Discussion

### Choosing the “cross technique” as a comparative study

LESS is the most rapidly developing minimally invasive surgery in recent years and has established advantages, including less invasiveness and better cosmetic effects. The main bottleneck problem of LESS is the tubular visual field and the collision of the instruments. Previous research has mostly focused on the improvement of instruments [8] to refine LESS, but there are few studies on the improvement of the surgical technique. The present study starts from other perspectives regarding the improvement of surgical techniques to optimize LESS using conventional laparoscopic instruments and equipment, which adjust the position of the surgeon, the layout of the instruments, and the operation technique. The chief surgeon stands by the head of the patient and holds two-hand parallel instruments that have independent fulcrums through the ports. To verify the feasibility and potential advantages of the new “chopstick technique,” we will compare it with the widely used “cross technique.” This study (CCLEH) will be the first prospective randomized controlled trial to compare and analyze the clinical characteristics of the two LESS techniques under the control of confounding factors.

### Choosing total hysterectomy as the research procedure

Most of the similar research related to the new surgical method used in the field of gynecology has chosen total hysterectomy as the research object. Jason D Wright [18] enrolled 264,758 cases of total hysterectomy for benign diseases in women and conducted a cohort study to compare robot-assisted laparoscopic technology and traditional laparoscopic technology. Robot-assisted laparoscopic total hysterectomy has similar complications to traditional laparoscopic surgery but higher costs. Tae-Joong Kim [19] carried out a multicenter prospective randomized controlled study to compare the surgical outcomes of multiport and single-port laparoscopic hysterectomy and demonstrated that single-port laparoscopic surgery is similar to multiport laparoscopic surgery in terms of the conversion rate and complication rate. From traditional laparoscopy to vNOTE surgery development in gynecology, JF Baekeland first initiated a prospective randomized trial comparing the characteristics of the two surgical methods in 2018 [20], exploring the application of vNOTE. The study still selected total hysterectomy as the research object to research the surgical efficacy of two LESS techniques, the “chopstick technique” and “cross technique.”

Total hysterectomy is a staged representative gynecological operation. As a research object, it has the following advantages: (1) total hysterectomy is a standard treatment surgery for many benign gynecological diseases and can be carried out at many medical centers. The operation volume satisfied the sample size of clinical research. The number of completed operations exceeds the surgeons’ learning curves, eliminating the difference in the period of the learning curve. (2) The procedure of total hysterectomy is procedural and easy to master; additionally, it includes multiple delicate operation steps, such as exposure and electrocoagulation of the uterine artery and suturing of the stump, which can be used to evaluate surgical differences.

### Main research indicators and the control of confounding factors and bias

The criteria of a newly successful technology are easy to master, preferred by surgeons and ultimately benefiting patients. However, these evaluation indicators are more complicated, with some being subjective, and some are difficult to evaluate. At present, the superior indicator for the evaluation of surgical techniques is operation time, which is also an objective evaluation parameter for differences in surgical techniques. The reduction in operation time can also indirectly reflect the benefits to patients. Therefore, this study selected operation time as the primary endpoint and other indicators, such as the fatigue of surgeons, as secondary indicators.

The size of the uterus affects the difficulty of total hysterectomy, which is the main confounding factor of the influence of the surgical technique on the operation time. According to the size of the uterus, we set up a stratified grouping study to eliminate the influence of uterine size as a confounding factor of the study. The intraoperative separation of adhesions is another influencing factor of surgical difficulty and a major research indicator in LESS hysterectomy. The study excluded cases that may cause severe intraoperative adhesions and added surgical time classifications during the operation: the single-port establishment time, pelvic-abdominal adhesion release time, total hysterectomy time, specimen removal time, and stump suture time, which eliminated research bias.

This study limits the qualifications of surgeons to those who have mastered both technical methods and have completed more than 40 LESS operations, thereby overcoming the learning curve of the new technology. Surgical videos of each operation are retained, and these will be evaluated by the clinical trial data monitoring committee (DMC). Above all, the research results will have better validity and universality.

## Limitations

This study will be a clinical study conducted at a single center, and the experience of doctors in the center may limit the universality of the conclusions of this study. RCT research will be conducted under ideal experimental conditions, and the surgeons are proficient in both surgical skills. It cannot be ignored that these two LESS technologies are used at different frequencies in a real environment, and the validity of the research results will be interpreted cautiously. Multicenter prospective cohort studies can increase the credibility of the results. However, this also increases the confounding factors of differences in surgeons’ operation skills.

### Implications for clinical practice

This study will be a pilot study comparing the application of the chopstick technique and the cross technique in LESS. Our center has carried out an observational study of LESS radical hysterectomy of cervical cancer with the chopstick technique and has reported its feasibility and preliminary safety. This is the first study to compare the efficacy of the two techniques in LESS: the chopstick technique vs. the cross technique. It is expected to accumulate safety data on the new techniques and provide information for sufficient surgical training in standard daily surgical practice techniques for use in women who must undergo hysterectomy. The present study will be a stage 2b clinical investigation based on the terminology used in the Idea, Development, Exploration, Assessment, Long-term study (IDEAL) collaboration. This research is a necessary opening for the long-term and scientifically rigorous evaluation of complex surgical interventions with different LESS techniques.

### Ethics and dissemination

The experiment will be carried out in accordance with this research protocol. We will fully inform the participants about the possible adverse reactions, risks, discomfort, and inconvenience, including the related treatment, in addition to encouraging rigorous compliance with the rule of voluntary participation, and providing a detailed patient information document for participants. All records will be stored in a particular secure storage area that has limited access to ensure participant confidentiality.

Major modifications of the protocol will be completed by the project applicant and main investigator together and resubmitted to the Ethics Committee for final review. After revising the program, there will be researcher trainings. If participants are involved, we will reobtain informed consent.

After the study, the related dataset will be uploaded to the corresponding public database by digital coding which hides patient personal information. The research results will be presented in peer-reviewed journals and at scientific conferences. All trial investigators will contribute to authorship.

## Current trial status

The CCLEH trial protocol is registered as ChiCTR2000040843 in the Chinese Clinical Trial Registry. The research protocol and informed consent documents were approved by the Ethics Committee of the First Affiliated Hospital of Army Medical University, PLA, on June 16, 2020. The first patient was recruited on July 1, 2021.

## Supplementary Information

**Additional file 1.**
